# Mental health and climate change – a developmental life course perspective

**DOI:** 10.1192/j.eurpsy.2021.619

**Published:** 2021-08-13

**Authors:** F. Vergunst, H. Berry

**Affiliations:** 1 Public Health, University of Montreal, Montréal, Canada; 2 Australian Institute Of Health Innovation, Macquarie University, Sydney, Australia

**Keywords:** disasters, climate change, developmental psychopathology, child and adolescent mental health

## Abstract

**Introduction:**

Climate change is a major global public health challenge that will have wide ranging effects on human psychological health and wellbeing through the increased incidence of acute (e.g., storms, floods, wildfires), sub-acute (e.g., heat stress, droughts, lost agricultural yields) and long-term stressors (e.g., changes to landscapes and ecosystems). Children and adolescents are particularly at risk because of their rapidly developing brain, vulnerability to disease and limited capacity to avoid or adapt to climate change-related threats and impacts. They are also more likely to worry about climate change impacts than any other age group.

**Objectives:**

To produce a new conceptual framework that describes climate change-related threats to youth mental health from a developmental life course perspective.

**Methods:**

We critically review and synthesis literature documenting the pathways, processes and mechanisms linking climate change to increased mental health vulnerability.

**Results:**

We show that climate change-related threats can additively and interactively increase psychopathology risk from conception onwards, that these effects are already occurring and that they constitute an important threat to mental health and therefore human capital worldwide. We then argue that birth cohort studies are uniquely positioned to examine climate change-related threats and that incorporating relevant measures into existing and planned birth cohorts is a matter of social justice and crucial long-term investment in mental health research.

**Conclusions:**

Climate change is affecting the healthy psychological development of children and these risks are increasing worldwide. New theoretical and empirical work is urgently needed so that threats can be tracked and mitigated.

